# Multiple Sclerosis in Malaysia: Demographics, Clinical Features, and Neuroimaging Characteristics

**DOI:** 10.1155/2013/614716

**Published:** 2013-12-26

**Authors:** S. Viswanathan, N. Rose, A. Masita, J. S. Dhaliwal, S. D. Puvanarajah, M. H. Rafia, S. Muda

**Affiliations:** ^1^Department of Neurology, Kuala Lumpur Hospital, Jalan Pahang, 50586 Kuala Lumpur, Malaysia; ^2^Department of Radiology, Kuala Lumpur Hospital, Jalan Pahang, 50586 Kuala Lumpur, Malaysia; ^3^Autoimmune Unit, Allergy and Immunology Research Center, Institute for Medical Research (IMR), Jalan Pahang, 50586 Kuala Lumpur, Malaysia; ^4^National University of Malaysia, Jalan Ya'acob Latif, Bandar Tun Razak, Cheras, 56000 Kuala Lumpur, Malaysia

## Abstract

*Background*. Multiple sclerosis (MS) is an uncommon disease in multiracial Malaysia. Diagnosing patients with idiopathic inflammatory demyelinating diseases has been greatly aided by the evolution in diagnostic criterion, the identification of new biomarkers, and improved accessibility to neuroimaging in the country. *Objectives*. To investigate the spectrum of multiple sclerosis in Malaysia. *Methods*. Retrospective analysis with longitudinal follow-up of patients referred to a single tertiary medical center with neurology services in Malaysia. *Results*. Out of 245 patients with idiopathic inflammatory demyelinating disease, 104 patients had multiple sclerosis. Female to male ratio was 5 : 1. Mean age at onset was 28.6 ± 9.9 years. The Malays were the predominant racial group affected followed by the Chinese, Indians, and other indigenous groups. Subgroup analysis revealed more Chinese having neuromyelitis optica and its spectrum disorders rather than multiple sclerosis. Positive family history was reported in 5%. Optic neuritis and myelitis were the commonest presentations at onset of disease, and relapsing remitting course was the commonest disease pattern observed. Oligoclonal band positivity was 57.6%. At disease onset, 61.5% and 66.4% fulfilled the 2005 and 2010 McDonald's criteria for dissemination in space. Mean cord lesion length was 1.86 ± 1.65 vertebral segments in the relapsing remitting group as opposed to 6.25 ± 5.18 vertebral segments in patients with neuromyelitis optica and its spectrum disorders. *Conclusion*. The spectrum of multiple sclerosis in Malaysia has changed over the years. Further advancement in diagnostic criteria will no doubt continue to contribute to the evolution of this disease here.

## 1. Introduction

Multiple Sclerosis (MS) is an uncommon disease with an estimated prevalence of one to two per 100,000 population in multiracial Malaysia with its Malay, Chinese, Indian, and indigenous racial groups [1–3]. Previous studies have highlighted several features such as rare familial inheritance and higher prevalence amongst females and those of Chinese descent. Clinically, these studies also reported optico-spinal preponderance, paroxysmal tonic spasms, early blindness, severe disability, and low incidence of oligoclonal bands and cord lesions of more than 3 vertebral segments (VS) on the magnetic resonance imaging (MRI) of the spine [1–3]. Since 1988, very little has been published on the demographics and clinical characteristics of patients with classical western type multiple sclerosis (MS) in Malaysia.

There have been a number of revisions to the diagnostic criteria for MS over the last 15 years (2001, 2005, and 2010) while the revised Wingerchuk criteria (2006) incorporates the anti-aquaporin 4 antibody (anti-AQP4 Ab) in the diagnosis of neuromyelitis optica (NMO) and its spectrum disorders (NMOSD) [4–10]. The evolution in diagnostic criteria and the recent accessibility to anti-AQP4Ab testing prompted re-evaluation of the changing spectrum of MS here in Malaysia.

## 2. Method and Materials

### 2.1. Objectives

The primary objective of this study was to characterize patients with multiple sclerosis in Malaysia in terms of demographics, clinical features, laboratory characteristics and neuroimaging of the brain and the spine.

In order to achieve this, every effort was made by incorporating clinical and paraclinical evidence such as oligoclonal bands, the anti-AQP4 antibody test and neuroimaging in excluding patients with neuromyelitis optica (NMO) and its spectrum disorders (NMOSD) as well as other idiopathic inflammatory demyelinating disorders (IIDDs). A comparison was drawn between these two groups (MS and NMO/NMOSD) with regard to demographics and MRI of the spine.

### 2.2. Patient Criteria

This was a retrospective, observational study with longitudinal follow-up of patients who had presented to the Neurology Department, Kuala Lumpur Hospital (Kuala Lumpur, Malaysia) from 2008 to 2013. Consecutive patients presenting to the Neurology department with IIDDs (all who consented were tested for Anti- AQP4 antibody) were identified from the Demyelinating Diseases' Registry^(MREC 10503)^ and included in this study.

These patients were diagnosed with clinically definite multiple sclerosis [4, 6, 7] or McDonald's multiple sclerosis (MS) [6, 7] based on McDonald's 2005 and 2010 criteria. Other patients with IIDDs were diagnosed with neuromyelitis optica based on the Wingerchuk 2006 [8–10] criteria (Table 1).

The remaining patients were diagnosed with neuromyelitis optica spectrum disorder and included those patients with single episode or recurrent optic neuritis, single episode or recurrent longitudinally extensive transverse myelitis with cord lengths of more than 3 vertebral segments and patients with brain involvement at onset of disease with demyelinating lesions not typical of multiple sclerosis who were anti-AQP4 antibody positive.

Patients with relapsing short cord myelitis (<3 vertebral segments VS) with brain MRI undeclared as MS with or without oligoclonal band positivity and anti-AQP4 antibody negativity were diagnosed initially with spinal multiple sclerosis until they developed anti-AQP4 positivity or brain MRI declared itself as NMOSD-like on follow-up.

Patients with opticospinal presentation with cord lesions less than 3 VS with normal brain MRI or few nonspecific small subcortical brain lesions not in keeping with MS were diagnosed with opticospinal multiple sclerosis (OSMS) [11].

Those patients with IIDDs with brain involvement at onset of disease as described by Kim et al. [12], atypical of multiple sclerosis were diagnosed as neuromyelitis optica spectrum disorder with brain involvement. These included subjects who were initially negative for anti-AQP4 antibody but on retesting were positive or alternatively remained negative. Lastly, the term clinically isolated syndrome was applied to patients who presented with first episode of demyelination (not NMO/NMOSD-like) with negative anti-AQP4 antibody (Table 1).

All other IIDDs not meeting the above criteria including patients with ADEM, single episode optic neuritis or transverse myelitis undeclared as MS or NMO/NMOSD (with MRI_brain negative, anti-AQP4 negative), unclassifiable recurrent optic neuritis, idiopathic or post infective transverse myelitis and non-IIDDs were not included. All these latter patients were anti-AQP4 antibody negative. Furthermore, in this group the cerebrospinal fluid oligoclonal bands were negative and where positive there were bands in the corresponding serum.

The racial distribution, sexual preponderance, clinical features with expanded disability status scale at onset of disease, and neuroimaging features (MRI of brain as well as the distribution, type and, length of spinal cord segment involved) in patients with MS were analysed.

All patients' case notes and neuroimaging, both at onset of disease and on follow-up (when available), were reviewed by a single neurologist (SV). The initial diagnosis of all patients was reviewed and when necessary re-evaluated based on current criteria.

### 2.3. MRI

Brain and spine MRIs were done at onset and on follow-up using a 1.5 Tesla machine. When patients were referred from another hospital for an opinion, every effort was made to obtain the first MRI of the brain and spine done when the patient presented with the first symptom or symptoms or the first MRI brain and spine done when the patient first presented to that particular referring hospital. Axial and sagittal views of T1- and T2-weighted images (WI), fluid-attenuated inversion recovery (FLAIR) images, and T1 pre- and postgadolinium WI were obtained. These were carried out at first presentation, within 3 months and annually, or if there were relapses. These brain and spine MRIs were reviewed by a single neuroradiologist (NR) in consultation with the single neurologist, SV.

### 2.4. Statistical Analysis

Data was analysed using SPSS version 16 software, (SPSS Inc, Chicago, IL, USA) looking at descriptive data, means, medians, percentages, and standard deviations. Nonparametric data was evaluated using the Wilcoxon signed rank test.

### 2.5. Method of Anti-AQP4 Antibody Testing

The anti-AQP4 test was conducted on a cell line at the Autoimmune Unit, Allergy and Immunology Research Centre, Institute for Medical Research, Kuala Lumpur. Anti-AQP4 antibodies (AQP-4) were determined in the serum of the patients by cell based indirect immunofluorescence assay (EUROIMMUN AG, Lubeck, Germany) which utilizes fixed, AQP4-transfected human embryonic kidney (HEK) cells on slides as an antigenic substrate.

Biochip slides containing AQP-4 transfected cells and nontransfected cells (EU-90) were incubated with diluted patient samples. In the case of positive reactions, specific antibodies of the classes of IgA, IgG, and IgM will bind to the antigens. In a second step, the attached antibodies are stained with fluorescein-labelled anti-human antibodies and made visible with the fluorescence microscope.

## 3. Results

A total of 245 patients with idiopathic demyelinating disease were identified with 104 patients categorized as MS and 77 patients as NMO/NMOSD. 26 patients were identified with acute demyelinating encephalomyelitis (ADEM) and undeclared as NMO or MS were, 7 patients with a single episode of optic neuritis, and 20 patients with single episode, transverse myelitis (all of whom had MRI of brain and or spine negative for MS or NMOSD). Eight patients had unclassified, single episode demyelinating brain disease while 3 other patients had relapsing optic neuritis of unclassified type (MRI of brain and spine negative for MS,NMO, or non-IIDDs).

## 4. Demographics

### 4.1. Patients with Multiple Sclerosis

Out of 104 patients, females made up 83% and males 17%, giving a female to male ratio of 5 : 1. Mean age at onset was 28.6 ± 9.9 years with a mean duration of illness of 6.41 ± 5.23 years.

Malays were the predominant racial group affected with 55/104 (52.9%), followed by the Chinese, 19/104 (18.3%) and Indians, 28/104 (26.9%), all of whom were from West Malaysia as well as 2/104 (1.9%) subjects of indigenous origin from Sabah and Sarawak in East Malaysia (Table 2). Interestingly, in the subgroup analysis of NMO/NMOSD patients there were more Chinese with NMO/NMOSD rather than MS, that is, Malays 38/77 (49.4%), Chinese 32/77 (41.6), and Indians 7/77 (9.1%).

### 4.2. Diagnosis and Laboratory Investigations

At the onset of disease, 64/104 (61.5%) and 69/104 (66.4%) fulfilled McDonald's criteria (2005, 2010) [6, 7], respectively, for dissemination in space (DIS) on neuroimaging. On subsequent follow-up of consecutive patients, 60/104 (56.7%) were classified with 2 attacks and objective clinical evidence of two lesions and 26/104 (25.0%) with 2 or more attacks and objective clinical evidence of one lesion with brain MRI demonstrating DIS consistent with MS and positive oligoclonal bands (OCB) in cerebrospinal fluid (CSF). Four patients (3.8%) had one attack with objective clinical evidence of two or more lesions with evidence of dissemination in time (DIT). Seven patients (6.7%) had one attack with objective clinical evidence of only one lesion and MRI brain in keeping with DIS and DIT, that is, the clinically isolated syndrome with clinically definite multiple sclerosis (CDMS) on MRI. About 3/104 (2.9%) of patients had a progressive course from the beginning.

Five patients had relapsing myelitis with short cord segments less than 3 VS but only 4/104 (3.8%) were included in the final analysis as one tested positive for anti-AQP4 antibody. These four patients' brain MRIs did not fulfil Swanton's [7] criteria for DIS/DIT for MS. The one patient who was positive had recurrent short cord myelitis on spinal MRI with paroxysmal tonic spasms and brain MRI with nonspecific small white matter lesions not in keeping with MS. So symptomatically, there were already warning signs of NMO. Three patients had pure relapsing optic neuritis with CDMS on MRI brain.

Of the 10 patients initially diagnosed with optico-spinal recurrents based on clinical criteria [11], 8 patients tested positive for anti-AQP4 antibody and were subsequently excluded from the MS group. Two patients with optico-spinal presentation were negative. Of the 8 patients who tested positive, retrospective analysis of their brain and spine MRI's obtained at onset of disease revealed 2 patients having cord lesions between 2 and 3 vertebral segments and the remainder 6 patients having longitudinally extensive cord lesions. The 6 patients were initially diagnosed as MS as their MRI spine at onset of disease was not available. The MRI of brain and spine reviewed at the first visit was taken during convalescence when the cord lesions had become patchy, short segment interrupted lesions. The 2 patients who were negative for anti-AQP4 antibody had cord lesions of ≤3 vertebral segments and brain MRI's showing an asymptomatic single subcortical gadolinium enhancing lesion and nonspecific subcortical brain lesions, respectively.

## 5. Clinical Features

The optic nerve (37/104, 35.6%) and the spinal cord (26/104, 25.0%) were the commonest sites of involvement at onset. However, this did not imply that patients had pure opticospinal presentation at onset as their MRI of brain fulfilled criteria for MS. This was followed by symptoms localized in the subcortical white matter (22.1%), brainstem (9.6%), and cerebellum (7.7%). At first relapse, cord presentation 33/104 (31.7%) appeared more common than optic nerve involvement (20/104), followed by symptoms localized to the subcortical white matter, brainstem and cerebellum. One patient presented with seizure at first relapse.

At onset, the common presenting complaints were monoparesis (6/104, 5.8%), hemiparesis (7/104, 6.7%), paraparesis (15/104, 14.4%), sensory complaints (21/104, 20.1%), blurring of vision, scotomas or loss of vision with or without pain around the eye (37/104, 35.6%), unsteady gait (7/104, 6.7%), vertigo(3/104, 2.9%), double vision(3/104, 2.9%), internuclear ophthalmoplegia (3/104, 2.9%), facial pain(1/104, 0.96%) due to trigeminal neuralgia, and cognitive impairment (1/104, 0.96%). A disseminated onset was seen in 15.4% of patients. Paroxysmal tonic spasms were uncommonly seen in only 17.3% of patients.

Out of the 104 patients with MS, the relapsing-remitting course of disease was seen in 89.4%, secondary progressive multiple sclerosis in 5.8%, and primary progressive disease in 2.9%. After 10 years of disease nineteen patients had become secondary progressive with mean expanded disability status scale (EDSS) of 6.59.

The mean number of relapses was 2.98 ± 2.46 per year from disease onset with an annual relapse rate of 0.87 ± 0.75. The mean EDSS at disease onset was 2.71 ± 1.84 for the entire cohort regardless whether relapsing or progressive, and mean EDSS after 13 years was 3.32 ± 2.71. Mean duration of diagnosis was 15.6 (±15.3) months. Family history was positive in five patients (5%). In one family, the mother and daughter both had MS, and the father had seropositive myasthenia gravis.

The majority of the patients in our series are still ambulating independently, that is, 75.0%, and 13.5% are walking with support, 3.8% are wheelchair-bound, and only 7.7% are nonambulatory and bedbound. In terms of visual acuity in the best eye, 90.4% had visual acuity of above 20/30 and 5.8% between 20/30 and 20/200, and the remainder reported visual acuity of less than 20/200 to no perception of light.

## 6. Neuroimaging of the Brain and Spine

MRI's of brain and spine were carried out at disease onset, on follow-up (once or twice a year), at relapse, or if the disease had a more aggressive course, in order to assess the lesion load. At disease onset 13/104 (12.5%) had cord lesions of one vertebral segment (VS) or less, 40/104 (38.5%) had cord lesions of more than one vertebral segment but ≤3 VS. Twenty two subjects (21.2%) presented with multiple, ill defined, patchy, short segments between 1 and 3 VS which when added up came up to >3 VS and were located sometimes close together, almost coalescing or separated along the cord (all these patients were anti-AQP4 negative) and 1.9% (2/104) presented with multiple well-defined short segments which when added up were <3 VS. No lesion was found in 19.2% (20/104) of patients, and MRI spine was not carried out in 7 patients.

The mean cord lesion length was 2.06 ± 1.92 vertebral segments (VS) when all patients either in the relapsing-remitting, or progressive phase of disease were included at disease onset. When only patients with relapsing-remitting course were included the mean cord lesion length at onset was 1.86 ± 1.65 VS. In the majority (65.4%) of patients the lesion was located in the periphery of the cord (postero-laterally or laterally). However, a few had central gray matter involvement (8.6%) at the maximum width of the cord lesion (Table 3).

**Figure 1:**
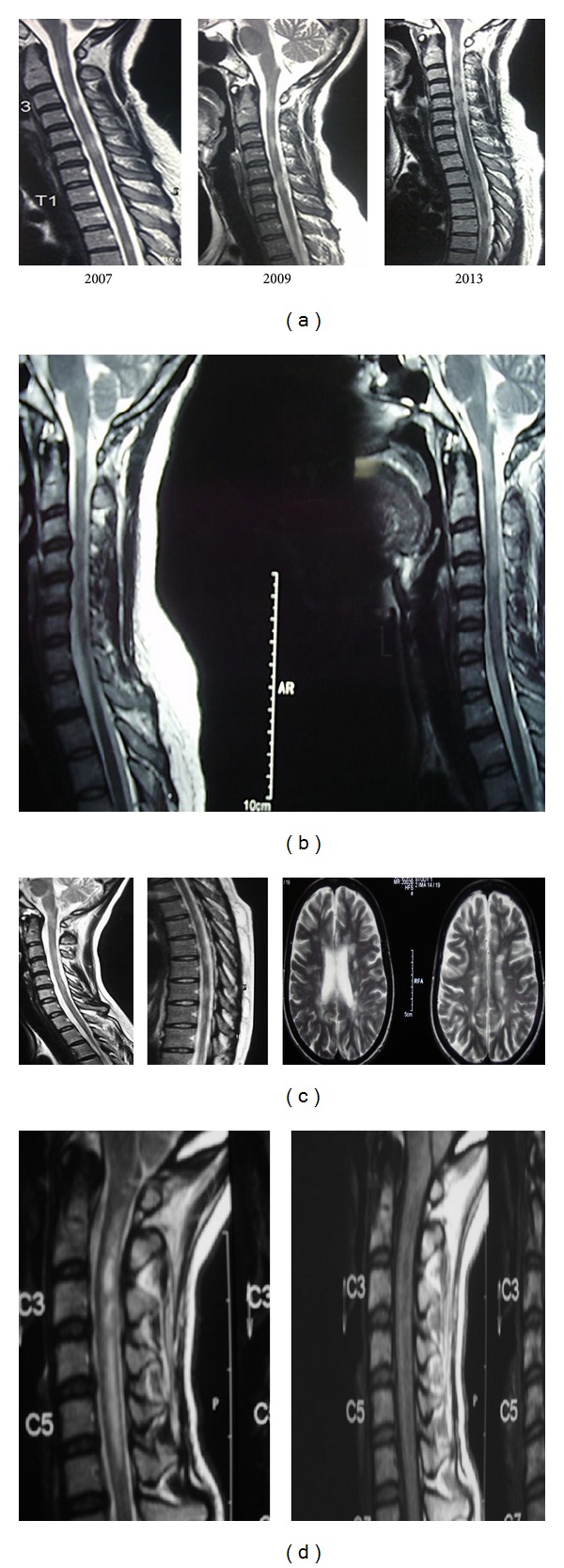
(a) Sagittal T2WI of the spine showing coalescing of multiple short segment lesions in a patient with relapsing-remitting multiple sclerosis at onset of disease and on follow-up once she entered the progressive phase of disease. (b) Sagittal T2WI of the spine showing a relapsing-remitting patient with multiple sclerosis with a short segment lesion superimposed on a longer patchy lesion at onset of disease. (c) Sagittal T2WI of the spine showing patchy ill-defined lesions more than 3 vertebral segments involving the entire cervicothoracic cord and axial T2WI of MRI brain with clinically definite multiple sclerosis in a patient with secondary progressive disease. (d) Sagittal T2WI (left) of the cervical spine showing a longitudinally extensive cord lesion with edema and corresponding T1WI (right) with T1 hypointensity within the cervical cord in a patient with neuromyelitis optica.

The commonest site for cord involvement was the cervical cord in 42/104, 40.4%, and cervicothoracic cord in 24/104, 23.1%. The thoracolumbar and the cervicothoracic, and lumbar cord were also involved. None of these patients had longitudinal contiguous or longitudinal linear lesions at disease onset. Twelve out of 25 patients with MS in the secondary progressive stage of disease and four with aggressive disease tended to have longer cord lesions of more than 3 VS on follow-up which was not there at disease onset (Figures 1(a), 1(b), and 1(c)).

## 7. Laboratory Investigations

Cerebrospinal fluid studies for oligoclonal bands (OCB) (isoelectric focusing method) were obtained in 66 patients of which 38 (57.6%) were positive. CSF pleocytosis was rare and ranged between 5 and 10 lymphocytes/mm [3]. Thirty-eight patients refused to have a lumbar puncture due to the social taboos associated with the procedure, patient related fears, inaccessibility to the test and when the treating neurologist felt it was not necessary. The visual evoked and somatosensory potentials were abnormal in 48% and 23.1%, respectively. Only 4 patients had positive antinuclear antibodies (ANA) of between 1 : 40 and 1 : 80 titers (Table 2).

All patients with multiple sclerosis were tested for anti-AQP4 antibody. All were negative except for 8 patients who prior to recategorization by the neurologist SV had pure optic nerve and spinal cord presentation. Of these 8 patients, 4 had unilateral blindness and severe myelitis which was relapsing in nature. MRI of brain showed nonspecific white matter lesions not fulfilling Swanton's criteria.

Retrospective analysis of records obtained during the first presentation revealed the MRI spine at presentation from a neighbouring hospital to have cord lesions between 2.5 and 3.0 VS in 2 of them, 4 and 5 VS in 4 of them and in the remaining 2 patients to have longitudinally extensive contiguous cord lesions involving the entire cervico-thoracic cord. All of them tested positive for anti-AQP4 antibody on longitudinal follow-up and were excluded from the final 104 patients. The misdiagnosis happened as the MRI of the spine at time of referral to our tertiary institution was not the first MRI at onset of disease. With time the cord lesion had become patchy and interrupted, and so initially they were diagnosed as multiple sclerosis. With reevaluation, these patients were included in the NMO cohort.

## 8. Differences between the MS Group and the NMO/NMOSD Groups

Out of the 77 NMO/NMOSD patients, the commonest initial presentation was that of myelitis followed by optic neuritis. In terms of demographics it was interesting to see more Chinese having NMO/NMOSD than MS, with a ratio of nearly 2 : 1 (NMO: MS) (*P* = 0.004). However overall, the racial distribution between the MS and NMO/NMOSD groups was similar. Females were again the predominant gender affected with a ratio of 6 : 1. With regard to neuroimaging, patients with NMO had longer cord lesions with mean cord lesions of 6.25 ± 5.18 VS and 1.1 number of cord lesions.

Patients with NMO and relapsing myelitis and NMOSD patients with brain involvement at onset had cord lesions which were longitudinally contiguous or linear, with cord atrophy, majority in the cervicothoracic region which predominantly involved the central gray matter or had holocord appearance. These changes were not seen or were rare in MS patients at onset of disease. Longer cord lesions in MS patient were seen if there was a more aggressive disease or later on in the course of the disease. Edema and T1 hypointensities were more commonly seen in the NMO/NMOSD groups but rare in MS patients at onset of disease; see Table 3, Figure 1(d). Ten patients with NMO/NMOSD had short cord lesions < 3 vertebral segments at onset of disease, and in these patients, close evaluation of their clinical scenario, CSF studies, anti-AQP4 antibody status, and, persistently absent or atypical brain lesions for MS should alert the clinician of the suspicion of NMO/NMOSD.

## 9. Discussion

In our study, the clinical spectrum of MS in Malaysia was found to have changed since the late 80s. The findings in this study also highlighted the similarities that still hold true over time such as the high female preponderance in MS. This female preponderance has been described in other parts of Asia, for instance, in northern Japan where the female to male ratio is 3.38 : 1, and in the west where the ratio has now increased to more than 2 [11, 13–16].

The young remained the commonest age group affected with a mean age at onset of 29 years, similar to Caucasian patients [1, 2, 15–19]. Familial occurrence has not been recognized before in Malaysia and was very rarely reported from the rest of Asia [1–3, 11]. However, in our study, 5% had either a first-degree or second-degree relative afflicted with MS.

Malaysia has a multiracial population where Malays and indigenous people (67.4%) are the predominant ethnic group followed by the Chinese (24.6%) and Indians (7.3%) [20, 21]. So it was not surprising to find the Malays being the predominant race affected by MS followed by the Chinese and Indians. This result, however, was a change in the racial distribution from previous reports [1–3]. For the first time too, we find indigenous groups from east Malaysia with multiple sclerosis which has not been reported before thus reflecting possible environmental effects which need investigation.

Rapid urbanization (71% in 2010 compared to 26.8% in 1970) and the increase in rural to urban migration by Malays may account for this change in demographics of MS in Malaysia [20, 21]. During the late 1950's, the Malays were more involved in agricultural activities and lived in rural areas with poorer accessibility to hospital facilities. In 1975, 11.2% of Malays lived in urban areas as opposed 44.7% of the Chinese and 30.7% of Indians. By 1991, the proportion of urban Malays had increased to 46.1% due to rural to urban migration [20, 21]. With urbanization and westernization, increasingly sedentary lifestyles (74% of the population spends time in sedentary activities) [22, 23], lack of exposure to sunlight, and the effect of environmental factors may have impacted on the development of MS in Malaysia. Several epidemiological studies have suggested a causal relationship between urbanization, environmental factors, interplaying with genetic susceptibility and development of multiple sclerosis [16–19, 23].

The relapsing-remitting course of disease remains the commonest type of clinical presentation. Progressive disease, primary or secondary, was seen in 8.7%. Ten years later 18.3% had become secondary progressive. This is unusual in Asia. Here the progressive course of disease has been reported to be rare [1, 24]. However these values are less than those seen in the west. This can be explained by the difficulty in identifying this group of patients by the history and the short follow-up period of less than 10 years. We also did not see as many patients presenting with CIS, and this could be due to patients seeking treatment later as the mean time of diagnoses was 15 months.

Mean EDSS at onset and on long term follow-up was much lower than previous reports [1–3]. Patients were less disabled with low mortality. Most were ambulating independently or with support. Visual outcome in one or both eyes which had previously been reported as poor was now much better on long term follow-up. This may be due to better accessibility to treatment, improved awareness, and careful exclusion of NMO/NMOSD patients from the current study group.

A large proportion of patients fulfilled DIS brain criteria by McDonald's 2005/2010 [6, 7] at disease onset. The type and distribution of the lesions in the brain were very much similar to western descriptions as we excluded non-specific and atypical brain lesions for MS [24–28]. This is comparable to recent reports from Taiwan, Thailand, and Korea where 58% and 50%, respectively, demonstrated DIS at disease onset [13, 14, 29, 30].

It was interesting to see how the MRI spine and its descriptions had changed over time. In the past, longitudinally extensive contiguous spinal cord lesions had been reported as a feature of MS and, this was thought to reflect severe cord involvement in Asia [3, 11, 29, 31]. Now, since we were applying the revised criteria for NMO and utilizing the anti-aquaporin 4 antibody for diagnosis, the majority of our patients had short segment cord lesions between 1 and 3 vertebral segments. Where patients had longer cord lesions, we found they actually had multiple short segments which were well defined or patchy as well as patchy interrupted longer segments which were not as well defined and certainly did not look like longitudinally extensive cord lesions seen in NMO. In some, well-demarcated short segments were superimposed on patchy long segments as time passed and so looked longer. In these patients we were fortunate to have their initial MRI spine at onset of disease for comparison which showed short segment lesions, and all of them were anti-AQP 4 antibody negative. These types of cord segments have been described by western authors. Here between 10% and 13% of patients with MS, respectively, had diffuse lesions more than 2 VS not of the LESCL variety [32, 33].

Some patients who went into the progressive phase had patchy longer cord lesions which initially were well-demarcated short segments but later on became longish when these short segments coalesced. Again it cannot be overemphasized how important the timing of the MRI and MRI at onset of disease is in evaluating these patients as cord lesions increase in number, fragment and coalesce with time [32, 33]. Furthermore, we felt some patients presented late accounting for their longer lesions.

The mean cord lesion length was 2.06 VS. This is shorter than earlier reports in Asia, that is, 3.6 ± 3.3 VS [31] and comparable with cord lengths reported from western figures even though we had included the cord lengths of all MS patients at varying stages of disease. Upon including only relapsing remitting patients we found the cord length at onset to be 1.86 ± 1.65 VS similar to western descriptions [31, 32]. Reports from Korea and Thailand recently described shorter cord lengths of 0.9 to 1.29 VS in MS [14, 30, 31].

In our series we had four patients presenting with cord lesions of between 1 and 3 VS with disease restricted to the spinal cord so-called “spinal multiple sclerosis” all of whom were anti-AQP4 antibody negative. Three were oligoclonal band positive. Since disease onset, these patients have not shown any lesions in the brain. Therefore they remain outside the current Swanton's criteria for MS and only longitudinal prospective follow-up will show whether these patients are true “pure spinal” entity within the MS spectrum as described by Malaysian authors before or otherwise [1, 2]. Furthermore, pure opticospinal presentation was not as common as before and could be explained by careful exclusion of the NMO/NMOSD groups.

Oligoclonal band positivity too (>50%) was much higher than previously described though still less than the western series. CSF pleocytosis was rare [1–3, 34, 35]. This despite the challenges associated with getting a lumbar puncture which describes the local cultural differences when dealing with local MS patients. Regionally recent studies also report higher rates [14]. One explanation may be the smaller sample size and issues with testing.

In conclusion, this study shows that the type of multiple sclerosis we are seeing in Malaysia is constantly changing with regard to racial distribution, clinical presentation, severity of disease, neuroimaging, and laboratory findings. With access to earlier MRI, better use of available diagnostic criteria and ancillary laboratory testing we were able to categorize patients a little better as it has tremendous impact on therapy. We acknowledge the limitations to the study such as small sample size, retrospective descriptive design, possible exclusion of true CIS cases, and short study follow-up and hope to address these issues in the future. We also hope to look closely at the differentiating features between different types of IIDDs especially NMO/NMOSD and the MS groups.

## Figures and Tables

**Table 1:** Showing the anti-AQP4 antibody, oligoclonal band, and spinal MRI results for patients with idiopathic inflammatory demyelinating disease.

Diagnosis (after incorporating anti-AQP4 antibody, oligoclonal bands, and cord lesion length)	Anti-AQP4 antibody		Cerebrospinal fluid for oligoclonal bands		Spine MRI	
	Positive	Negative	Positive	Negative	Cord lesion length ≤ 3 VS	Cord lesion length > 3 VS
Clinical Diagnosis						
MS						
CIS (MRI brain CDMS)	0	7	5	2	5 (in two no lesions)	0
CDMS	0	91	30	16	55	22
Opticospinal recurrent type of multiple sclerosis	8**	2	0	10	4	6*
Spinal multiple sclerosis	1^#^	4	3	2	5	0
NMO	31	4	1	43	2	33
NMOSD						
Single episode optic neuritis/relapsing optic neuritis	3	1	0	0	0	0
Single episode transverse myelitis/relapsing transverse myelitis	7	2	0	9	1	8
Brain lesions typical of NMOSD	6	3	1	8	4	5

Other IIDDs						
ADEM	0	26	1	25	8	18
Single episode optic neuritis (undeclared as NMO or MS, no brain/cord lesions)	0	7	0	7	0	0
Single episode transverse myelitis undeclared as NMO/MS	0	20	0	10	8	12
Unclassified single episode demyelinating disease of the brain (undifferentiated from NMOSD/ADEM/MS/non-IDDS)	0	8	0	8	0	0
Unclassified relapsing optic neuritis (MRI of brain and spine negative for MS or NMO/NMOSD)	0	3	0	3	0	0

MS: multiple sclerosis; NMO: neuromyelitis optica; NMOSD: neuromyelitis optica spectrum disorder; VS: vertebral segments.

*Six patients initially diagnosed with opticospinal variants of Multiple sclerosis upon reviewing first MRI at onset of disease were found to have longitudinally extensive cord lesions.

**Eight patients reclassified as NMO.

^#^Reclassified as NMOSD.

**Table 2:** Showing the clinical features of multiple sclerosis patients in Malaysia. (A subgroup analysis of demographics in NMO/NMOSD patients was also included.)

	*N* = 104
Female : male/%	87 : 17 (83% : 17%) ( ratio: 5 : 1)
In the MS group: Malays : Chinese : Indians : others %	55/104 : 19/104 : 28/104 : 2/104 patients 52.9% : 18.3% : 26.9% : 1.9%
In the NMO/NMOSD group: Malays : Chinese : Indians : others %	38/77 : 32/77 : 7/77 : 0/77 patients 49.4% : 41.6% : 9.1% : 0%
Mean age at onset/years	28.6 ± 9.9
Mean duration of illness/years	6.41 ± 5.23
Site of involvement	At onset At first relapse
Optic nerve	37/104 (35.6%) 20/104 (19.2%)
Spinal cord	26/104 (25.0%) 33/104 (31.7%)
Subcortical	23/104 (22.1%) 32/104 (30.7%)
Brainstem and cerebellum	18/104 (17.3%) 19/104 (18.4%)
Mean number of exacerbations	2.98 ± 2.46
Annualized relapse rates/year	0.87 ± 0.75
Mean EDSS at onset	2.71 ± 1.84
Familial involvement	5/104 (5%)
Clinical course	
RRMS	93/104 (89.4%)
SPMS	6/104 (5.8%)
PPMS	5/104 (2.9%)
CSF (OCB %)	38/66 (57.6%)
Ambulating independently (current status)	78/104 (75%)
Visual acuity (20/30 and above) in one/both eyes	94/104 (90.4%)
ANA positivity (1 : 40 to 1 : 80)	4/104 (3.8%)
Initial brain MRI fulfills McDonald's 2005 criteria for dissemination in space McDonald's 2010 criteria for dissemination in space	64/104 (61.5%) 69/104 (66.4%)
Spinal multiple sclerosis	4/104 (3.8%)

MS: multiple sclerosis; ANA: antinuclear factor, RRMS: relapsing-remitting multiple sclerosis; SPMS: secondary progressive multiple sclerosis; PPMS: primary progressive multiple sclerosis; OCB: oligoclonal bands; EDSS: expanded disability status scale.

**Table 3:** Neuroimaging of the spine in patients with multiple sclerosis and neuromyelitis optica and its spectrum disorders in Malaysia.

Spinal cord findings	MS (*n* = 104)/%	NMO/NMOSD (*n* = 77)/%
Mean cord lesion length at onset for all clinical types/VS	2.06 ± 1.92	6.25 ± 5.18
Mean cord lesion length in RRMS patients	1.86 ± 1.65	—
Commonest site for cord involvement		
Cervical	42/104 (40.4)	28/77 (36.4)
Cervicothoracic	24/104 (23.1)	33/77 (42.9)
Thoracic	6/104 (5.8)	8/77 (10.4)
Thoracic and lumbar cord	1/104 (1.0)	1/77 (1.3)
Cervical/thoracic/lumbar cord (interrupted segments)	4/104 (3.8)	—
Whole spine		3/77 (3.9)
Location of the lesion		
Periphery of cord (lateral/posterolateral)	68/104 (65.4)	3/77 (3.9)
Central gray matter	9/104 (8.6)	36/77 (46.8)
Holocord	—	34/77 (44.2)
Length of cord lesions (vertebral segments-VS)		
1 VS or less	13/104 (12.5)	1/77 (1.3)
>1 VS to ≤3 VS (Single patchy/well defined lesion)	40/104 (38.5)	9/77 (11.7)
>3 VS (multiple ill-defined patchy short segments between 1 to 3 VS)	22/104 (21.2) (multiple ill-defined patchy short segments between 1 and 3 VS coalescing together)	63/77 (81.8) (longitudinally extensive or linear lesions with or without patchy interrupted segments)
Multiple well-defined short segments, <3 VS	2/104 (1.9)	0/77 (0)
No lesion	20/104 (19.2)	4/77 (5.2)
No scan done	7/104 (6.7)	—
Cord atrophy		
Yes	3/104/(2.8) (all with disease >10 years ± progressive phase)	26/77 (33.8)
No	74/104/(71.2)	47/77 (61.0)
T1 hypointensity		
Yes	2/104 (1.9)	17/77 (22.0)
No	75/104 (74.0)	56/77 (72.7)
Cord edema		
Yes	0/104 (0)	24/77 (31.2)
No	77/104 (74.0)	49/77 (63.6)

MS: multiple sclerosis; VS: vertebral-segments; RRMS: relapsing-remitting multiple sclerosis; NMO: neuromyelitis optica; NMOSD: neuromyelitis optica spectrum disorder.

## Abbreviations

(MS): Multiple sclerosis
(NMO): Neuromyelitis optica
(NMOSD): Neuromyelitis optica spectrum disorder
(IIDD): Idiopathic inflammatory demyelinating diseases
(VS): Vertebral segments
(OCB): Oligoclonal bands
(Anti-AQP4): Anti-aquaporin 4 antibody
(CDMS): Clinically definite MS
(DIS): Dissemination in space
(CSF): Cerebrospinal fluid
(DIT): Dissemination in time
(EDSS): Expanded disability status scale
(CIS): Clinically isolated syndrome.
